# Structural and magnetic properties of the Fe$_{3}$O$_{4}$ (110) surface

**DOI:** 10.1038/s41598-025-94599-6

**Published:** 2025-03-27

**Authors:** Anna Mandziak, José Emilio Prieto, Clara Gutiérrez-Cuesta, Miguel Ángel Niño, Michael Foerster, Juan de la Figuera, Paweł Nita

**Affiliations:** 1 Solaris National Synchrotron Radiation Centre, 30-392 Kraków, Poland; 2 Instituto de Quimica Fisica Blas Cabrera, CSIC, 28006 Madrid, Spain; 3 Alba Synchrotron Light Facility, 08290 Cerdanyola del Valles, Spain; 4 Faculty of Physics, Astronomy and Applied Computer Science, Jagiellonian University, 30-348 Kraków, Poland

**Keywords:** Magnetite 110, Magnetic domains, Photoemission microscopy, Information storage, Magnetic properties and materials

## Abstract

We have studied the (110) surface of Fe$_{3}$O$_{4}$ single crystals by means of X-ray Photoemission Electron Microscopy (PEEM) and Low-Energy Electron Microscopy (LEEM) to characterize its structural and magnetic properties. After sputtering and annealing a well defined surface was achieved. This preparation method resulted in a one-dimensional reconstruction formed by rows aligned in the [001] direction. By acquiring X-ray magnetic circular dichroism PEEM images at various azimuthal angles, the vector magnetization map of the (110) surface was obtained. Domains were observed with their magnetization aligned along the two $\langle 111\rangle$ type bulk easy axes which are in the (110) surface plane, featuring 180$^{\circ }$, 109$^{\circ }$, and 71$^{\circ }$ domain walls. The domain walls are of Néel type. Using the sum rules we estimated an iron spin and orbital magnetic moment of 3.4 $\mu _B$ and 0.6 $\mu _B$ respectively for the reconstructed surface. At the oxygen K edge we observe dichroic contrast of close to 1%, which is reversed relative of the contrast detected from octahedral iron in the L$_3$ edge.

## Introduction

Magnetite stands out as one of nature’s most striking magnetic materials. Humankind has been familiar with magnetized “lodestone”, specifically magnetite, since as early as 1 BCE in ancient Greece and even earlier in China. Being the oldest known magnetic substance, it has significantly influenced the realms of science and technology^1^. Its high conductivity, chemical stability, and high Curie temperature^2^ facilitate its use in magnetic applications. The field of paleomagnetism relies also heavily on its distinctive properties to record the earth magnetic field through the ages. The growing interest in magnetite for spintronic applications has been promoted by the prediction of half-metallicity in magnetite, implying that its conduction electrons might have a high degree of spin-polarization, although there have been conflicting experimental conclusions on this theme^3,4^. Exciting developments include the utilization of magnetite as a spin-injector source^5–8^ as well as the robustness of its magnetic properties even at sub-nanometer thickness^9^.

At room temperature (RT), magnetite exhibits a cubic inverse spinel structure with a lattice constant close to 8.40 Å. This structure can be envisioned as an arrangement of oxygen anions in a face-centered cubic lattice^10^. Iron atoms occupy partially the sites within tetrahedra (T$_{d}$ sites) or octahedra (O$_{h}$ sites) of oxygen atoms. T$_{d}$ sites are populated by Fe$^{3+}$ cations, whereas O$_{h}$ sites host both Fe$^{2+}$ and Fe$^{3+}$ cations. The ferrimagnetic configuration of magnetite originates from the dominant antiferromagnetic interaction between T$_{d}$ and O$_{h}$ sites. Magnetite exhibits a Curie temperature of approximately 850 K. The easy magnetization directions at room temperature are the $\langle 111 \rangle$ lattice directions. Upon cooling, the resistivity increases by two orders of magnitude crossing the Verwey transition^11^, the oldest known metal-insulator transition. The Verwey temperature T$_{V}$ is in the range 115 K to 125 K depending on the sample quality^12^. The crystal structure changes from cubic to monoclinic below T$_{V}$ and presents a complex charge order^13^. The presence of residual stresses in single crystal lowers T$_{V}$^14,15^.

Extensive research has been conducted on the (001) and (111) surfaces of magnetite both for single-crystals and thin films grown on various metallic and oxide substrates^7,16–21^. Previous studies have demonstrated that these surfaces can exhibit multiple terminations depending on the preparation method, with a predominant focus on (001) and (111) surfaces utilizing STM and scanning tunneling spectroscopy (STS) techniques^22–26^.

In contrast, limited attention has been dedicated to the investigation of the magnetite (110) surface. Studies on the (110) surface have primarily concentrated on its structural properties and its response to different preparation conditions. Scanning tunneling microscopy (STM) and low-energy electron diffraction (LEED) investigations have indicated a one-dimensional (1$\times$3) reconstruction for both bare crystals and thin films, highlighting a termination involving iron ions from two distinct magnetic sublattices^27–29^.

Although millimeter-sized magnetite crystals lack stable remanence in rocks due to their magnetic softness, their orientation and sectioning along specific crystal planes facilitate investigation. The interpretation of domain structures becomes more straightforward when the exposed surface contains one or more magnetic easy axes, as demonstrated by Chiba in 1964^30^. Magnetite presents at room temperature a negative first order cubic magnetocrystalline anisotropy (K$_{c1}<0$) giving rise to $<111>$ magnetic easy directions. The bulk domain walls belong to three fundamental types, involving changes of 180$^{\circ }$, 109$^{\circ }$ or 71$^{\circ }$ in the orientation of the magnetization inside a domain wall^31,32^. Consequently, a (110) surface, incorporating two different $\langle 111 \rangle$ directions, proves conductive to observe various bulk domain and domain wall configurations without the distortion produced by the shape anisotropy as is the case, for example, of the (100) surface^33,34^. Thus arrays of these wall types offer representative insights into the internal domain structure when the surface is the (110) one^35^.

We hereby present an analysis of the Fe${_3}$O${_4}$ (110) surface by synchrotron-based X-ray photoemission electron microscopy (XPEEM) providing insight into the structural, chemical, and magnetic properties of the material. While the magnetic properties of magnetite (110) were previously investigated applying methods which are not particularly surface-sensitive such as the Bitter technique ^35^ or magnetic force microscopy (MFM^36^), we resort to XPEEM which offers precise information about the surface magnetization with a spatial resolution reaching 20 nm. We focus our effort specially on the surface magnetization including domains and domain wall arrangement but also including a determination of the spin and orbital moments of the (110) surface prepared in ultra-high vacuum in the single crystal.

## Results

**Fig. 1:**
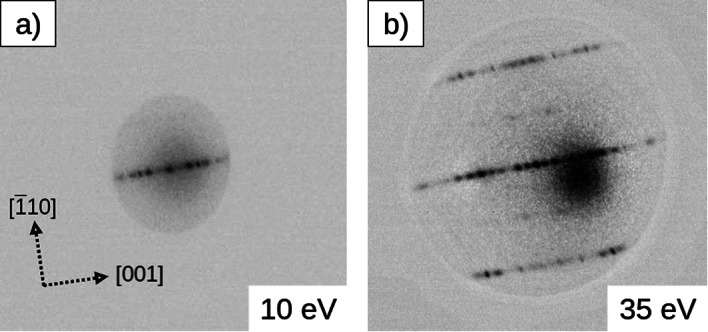
LEED of the magnetite (110) surface at 10 eV (**a**) and 35 eV (**b**). The orientation of the crystal is also shown in the first LEED pattern.

The best-quality Fe$_{3}$O$_{4}$ surface was obtained after sputtering and annealing at 800$^{o}$C, in agreement with previous work reported by Jansen et al^27,28^. This preparation method results in a (1 $\times$ 3) surface reconstruction that can be seen in the attached LEED images (Fig. 1). The LEED spots are consistent with a periodicity of 0.3 nm and 2.5 nm in the [$\overline{1}$10] and [001] directions, respectively. The second set of LEED spots suggest an additional 0.6 nm periodicity in the [$\overline{1}$10] direction. These spots were observed after annealing to slightly lower temperature around 710$^{o}$C. LEEM images taken at a clean magnetite surface do not show any characteristic features. According to previously obtained STM images, the Fe$_{3}$O$_{4}$ surface reveals rows in the [$\overline{1}$10] direction extended over hundreds of nanometers with a periodicity in the [001] direction of around 2.52 nm^29^. This is much lower than the nominal spatial resolution of the LEEM microscope (10 nm); for this reason we do not see any topographical features related to the surface reconstruction^37^.

In order to get insight on the surface chemistry of our sample, a series of XAS measurements was performed under different preparation conditions. Following the work of Jansen^27,28^ we have checked the effect of Ar ion bombardment. After a few cycles of sputtering (without annealing), the surface composition was oxygen deficient. The subsequent annealing caused an enhancement of the surface oxygen concentration and the restoring of the pristine composition^28^. However we have not observed any additional phases of iron oxides like hematite or maghemite on the surface after oxygen annealing. Thus, we can conclude that both methods for (110) surface preparation - annealing without and with molecular oxygen in the chamber lead to the same surface structure, even if the surface might present some differences in oxygen content. A similar result was reported by Ref. [28].

**Fig. 2:**
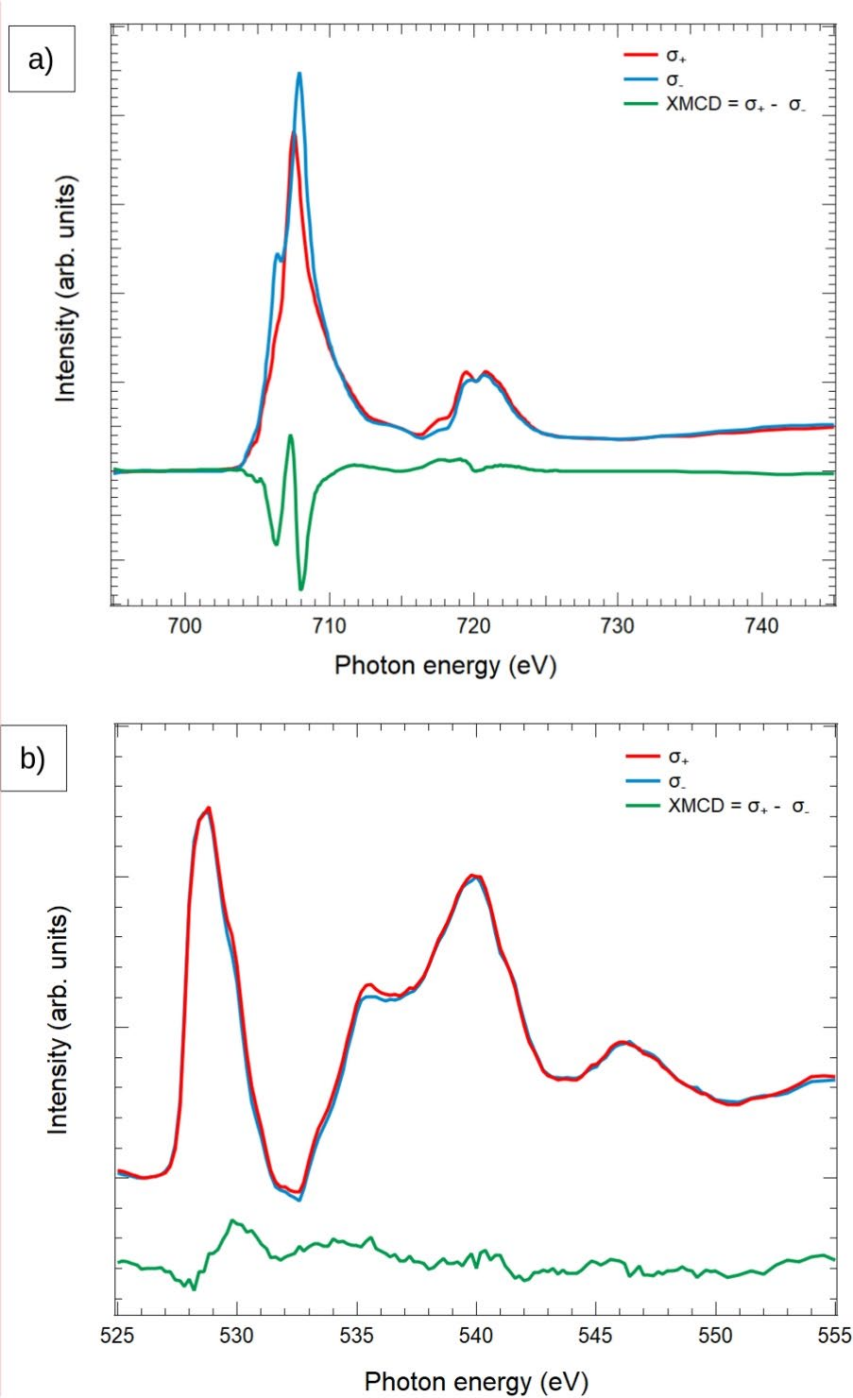
XAS and XMCD spectra of a single domain region of the magnetite (110) surface collected at (**a**) the Fe L$_{3,2}$ edges and (**b**) the O K edge.

To address the magnetic properties of the magnetite crystal, XAS spectra with two opposite circular polarizations of the X-ray beam have been acquired from a single magnetic domain (as determined by XAS images as described later). The XAS spectrum observed at the Fe L$_{3,2}$ edges is shown in Fig. 2a. It shows the typical features characteristic of magnetite^38–40^, arising from the Fe$^{3+}$ and Fe$^{2+}$ ions. Their presence is especially evident in the XMCD signal (bottom of Fig. 2a), where three peaks are visible, separated from each other by around 0.5 eV. These peaks are identified with iron cations located in octahedral (first and third) and tetrahedral position (second) within the spinel structure. The tetrahedral and octahedral cations have opposite moments because of superexchange coupling. The relative intensity of the three peaks has been used to estimate the populations of the three cations. However, there are limits to this interpretation, as the first peak also contain some contribution from the Fe$^{3+}$ cations. Nevertheless, the fact that the first negative peak is shallower than the second points to a Fe$^{3+}$-rich termination.

We have applied the L$_{2,3}$ sum rules^41^ to the Fe edges in order to estimate the magnetic orbital and effective spin moment. Their application requires knowledge of the number of holes, which in the case of iron has been calculated to be 13.5 per formula unit for bulk magnetite^42^. The effective spin moment comprises also the contribution of the dipole operator. However, this latter value is presumed to be small given the cubic symmetry of the magnetite lattice^43^. The determined magnetic spin and orbital moments are 3.4 $\mu _{B}$ and 0.6 $\mu _{B}$, respectively (yielding a total moment of 4 $\mu _{B}$ with a ratio of 0.18). However, we remark the large dispersion of experimentally determined spin and orbital moment for magnetite, depending on the research group even for the same type of material (bulk or thin film) and orientation (as a non exahustive list we refer the reader to^40,44–53^). This is in part likely to be related to the surface sensitivity of the XMCD measurement. This sensitivity is linked to the effective penetration depth of the x-ray beam, and to the mean free path of the secondary electrons which are detected. In our case, even for a shallow incidence angle of 16$^\circ$, the spectra should not be affected by self-absorption effects as the effective x-ray absorption length is then much larger than the electron escape depth^18^. The electron mean free path is still being discussed from the typical range of 5-10 nm predicted from the universal mean free path curve to much smaller experimental values below 1 nm^47^ for the small kinetic energies we use in the PEEM (start voltage of 1.25 V), in line to the values reported for other oxides such as CrO$_2$^54^. For such small values, the particular structure of the last few atomic layers is crucial. In the particular case of the (110) surface reconstruction, the latest proposal is composed of (111) faceting^29^. However, we currently lack a detailed atomic model of the surface reconstruction which includes the atomic charge distribution, and thus the number of holes per cation, in addition to the particular atomic magnetic ordering. Thus while we ascribe our result of a lower spin moment to the effect of the surface reconstruction and/or modified surface stoichiometry, a more detailed comparison will have to await a precise atomic model. We note that, for example, the (100) surface reconstruction^10^ presents a reduced net surface magnetic moment of the iron^18^, even if like in the (110) case, the surface is preferentially Fe$^{3+}$ terminated.

The XAS spectra taken at the oxygen K-edge are presented in Fig. 2b. It shows the typical features of spinel oxides^55–57^. They are interpreted in terms of the unoccupied density of states with O 2*p* character. The first double peak near 530 eV corresponds to the hybridization of the oxygen 2*p* states with the iron $t_{2g}$ and $e_g$ orbitals. The peaks at higher energy, near 537 eV, 540 eV and 547 eV correspond to the hybridization with the Fe 4*s* and 4*p* unoccupied states. Although a weak dichroism can be observed at the energy corresponding to the hybridization with the iron *d* bands (around 529 eV), as will be presented later, the problems with spectra normalization between the two polarizations in PEEM (where small changes in the x-ray beam position cannot be easily corrected), together with the small magnitude of the observed dichroism prevented us from measuring the full dichroic spectra.

We now focus on the surface magnetism of the (110) surface. We note that most of the characterization of magnetic domains at the surface of oriented bulk single crystals has been performed on the most compact surfaces: the (111), (100)^19,58^ and (110) orientations. At room temperature, the (110) surface stands out as the only low-index surface which has sets of $\langle$111$\rangle$ easy axes within the surface plane. This suggests that the patterns observed in this plane should be simpler and closely resemble the domain structure in the crystal interior, as proposed by Özdemir in their pioneering Bitter study of the (110) surface^35^, although this surface can also contain closure domains.

**Fig. 3:**
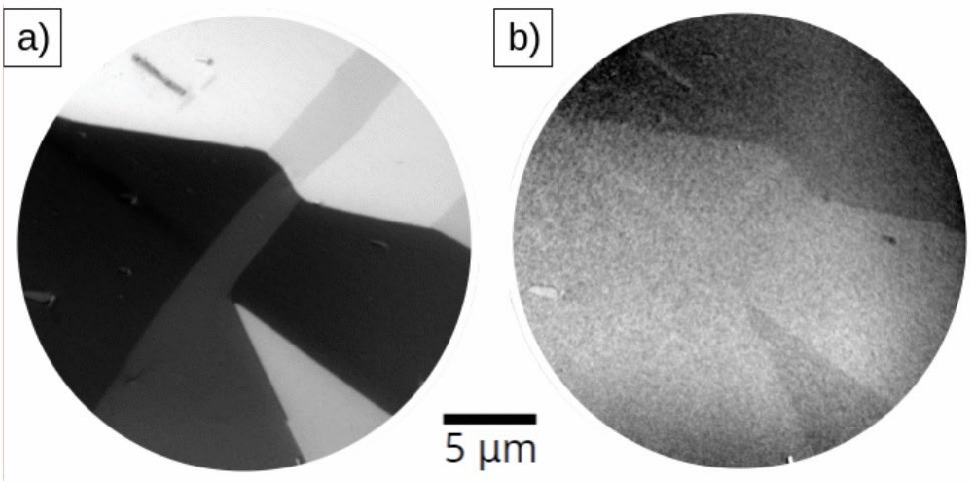
XMCD PEEM images acquired at (**a**) the Fe L$_{3}$ and (**b**) the O K edge on the magnetite (110) surface. Note the reversed contrast between the two images.

Figure 3 presents XMCD images from the same area collected at the Fe L$_{3}$ and O K edges, respectively. The particular energies are the energy of the lowest negative peak in the Fe XMCD spectra (708 eV), and the maximum of the O K edge (529 eV). The x-ray beam is coming from the right along the x-axis of the images (the XMCD images correspond to the component of the magnetization along the x-ray incidence direction)^43^. By acquiring LEED patterns in LEEM mode in the same region, we can also find the crystallographic axis of the surface relative to the magnetization directions. In this way, we have identified that the x-axis of the figure is 20$^{\circ }$ from the [001] direction and 110$^{\circ }$ from [$\overline{1}$10] direction, respectively. The contrast observed in Fig. 3a at the Fe edge is very strong ($\approx$ 30%) and four grey levels are detected (black, white and two shades of grey) corresponding likely to four magnetic domain orientations. Magnetic domains are observed to be completely unrelated to the topographic features. The magnetic contrast is also quite robust. We have measured this surface at two different UHV systems and even without cleaning the domains are observed directly after loading it into vacuum. However, we note that when the surface is not cleaned a clear LEED pattern is not observed.

**Fig. 4:**
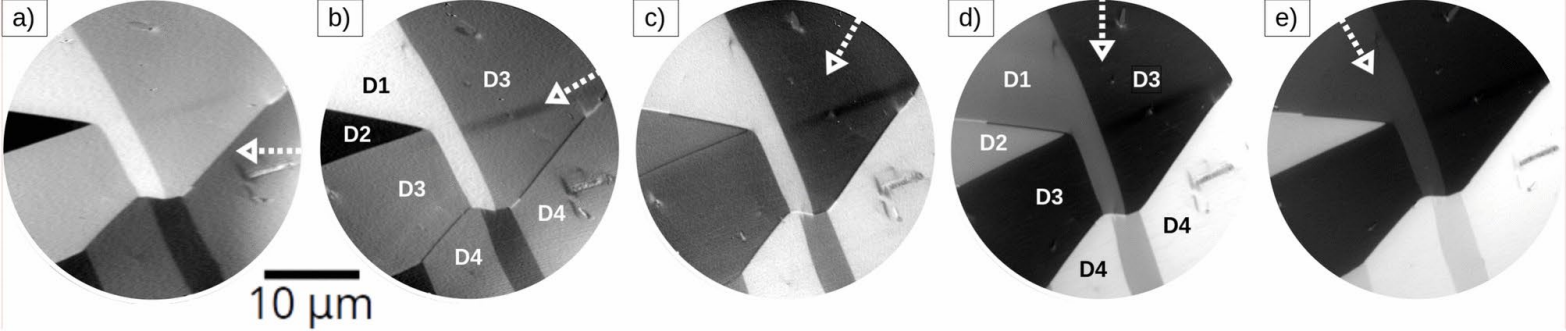
XMCD-PEEM images of the same area of the magnetite (110) surface, acquired at the Fe L$_{3}$ edge, at different azimuthal angles, of 0$^\circ$, 30$^\circ$, 60$^\circ$, 90$^\circ$ and 120$^\circ$. The images have been rotated back by the same amount in order to identify the same features. The incoming x-ray beam direction is marked by an arrow in each image.

At the oxygen edge, Fig. 3b, the contrast is much fainter, around 0.6%. While only two gray levels are clearly detected, another one can be observed by enhancing the contrast. Thus, we suggest we see the same domains in the iron and the oxygen edges. However, the contrast is reversed relative to the Fe one (Fig. 3a). This has been observed in several orientations of the x-ray beam, again observed reversed domains. The contrast in the oxygen K-edge likely arises from hybridization of the unoccupied oxygen *p* levels with the cation *d* levels, and would be expected to be aligned in the same orientation as the octahedral iron. However, this assumes a bulk surface termination. We suggest that the reconstruction observed by LEED is responsible for the inverted contrast. However, further work is planned to explore the detailed origin of such contrast.

To fully resolve the magnetic structure of Fe$_{3}$O$_{4}$(110) we have acquired Fe XMCD images at different azimuthal angles of the sample relative to the (fixed) x-ray beam. The images are shown in Fig. 4 and were collected for azimuthal angles of 0$^{\circ }$, 30$^{\circ }$, 60$^{\circ }$, 90$^{\circ }$ and 120$^{\circ }$. The images are then rotated back to present the same real space orientation but with a rotated x-ray illumination direction. As can be seen in that series at 30$^{\circ }$ in-plane incidence, the white and black domains (D1 and D2) are brighter and the other two are nearly the same gray (D3 and D4). This suggests that the surface magnetization in the latter domains (D3 and D4) is close to perpendicular to that incidence direction, i.e. along the 120$^{\circ }$ direction. And in the image acquired at 90$^{\circ }$, the situation is reversed: the mostly gray domains are now clearly black (D3) and white (D4), while the former white and black (D1 and D2) are nearly uniform gray. Likewise, this suggests that the domains in D1 and D2 are more closely oriented along the perpendicular direction to the incident x-ray beam in that image, i.e. along the 0$^{\circ }$ direction. This is in agreement with the angle between the two easy axis of the (110) surface, which is 109$^{\circ }$. Furthermore, the other images allow to observe clearly the domain walls between domains, indicating that they are Néel walls. This is in agreement with simulations of Bloch walls close to a surface in magnetite, which have been found to turn to Néel caps at the surface^59^. The meeting point of such two orientations along a domain wall is a Bloch line, of which several are observed in the bottom right domain wall observed more clearly in Fig. 4b.

For example, in Fig. 4 the domain walls (DWs) surrounding the triangular domain on the left side are observed. More details can be detected. In some of the domain walls, there is inversion of contrast when moving along the DW, such as along the long one that extends from the lower center region to the mid-right side, i.e. a Bloch point. Such points indicate that the DW at the surface do not select a single chirality. This is in agreement with similar observations of the DWs of the (100) magnetite surface^19^. However, in such case the domain walls are characteristically curved, the result of the competition of shape anisotropy and magnetocrystalline anisotropy near the surface region of the crystal.

**Fig. 5:**
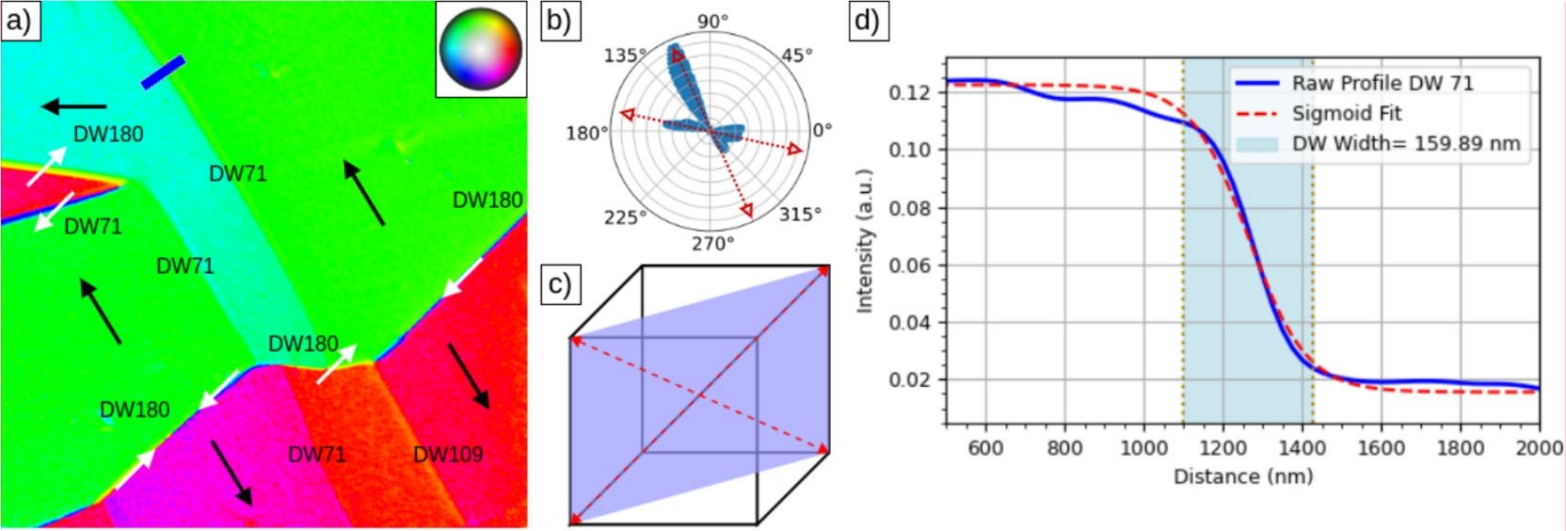
(**a**) Vector magnetization reconstructed from the overlapping region of three XMCD-PEEM images (from Fig. 4a, c, d)^61^. The magnetization directions are also indicated by arrows both in the domains and the domain walls. The type of each domain wall (DW71, DW109, or DW180) is labelled, (**b**) polar histogram of the in-plane magnetization presented in (**a**). In order to make the intensity more comparable, the square root of the histogram is plotted. Two arrows indicate the deduced easy axes directions, which correspond to the two in-plane $\langle$111$\rangle$ directions, (**c**) schematic of the observed axes relative to the bulk unit cell, (**d**) intensity profile across the domain wall, averaged over the marked rectangular region in Fig. 5a. The domain wall width is determined by fitting a sigmoid function to the extracted profile. From this fit, the width is estimated as 2.2$\delta$, where $\delta$ represents the characteristic transition width between two states, and the factor 2.2 corresponds to the 10–90% transition range of the sigmoid function.

By combining a minimum of three non-coplanar XMCD images the vector magnetization of the surface can be determined. More azimuthal components have been shown to increase the accuracy of the reconstruction, up to an optimum of five angles^60^. However, this requires a high accuracy to register the images rotated by different angles. Instead we have used the minimum number of three images required (corresponding to the 0$^\circ$, 60$^\circ$ and 120$^\circ$ angles)^61^. The reconstructed vector magnetization is presented in Fig. 5a where the in-plane magnetization is shown employing a color palette (see the inset at the top-right side of the image). In addition to the color map, arrows indicating the average magnetization in each domain, as well as the magnetization along domain walls are also indicated, as deduced from the color map as well as from the sequence of rotated images in Fig. 4 (the non-uniform illumination in the images in Fig. 4 give rise to a wrong magnetization identification in the lower right part of the image) . As a complementary way to visualize the magnetization orientation, we present in Fig. 5b a polar histogram of the distribution of in-plane magnetization values from the area presented in Fig. 5a. Overlapping on it are the two main easy axes which can be extracted from such histrograms. It clearly shows that the magnetization on the surface is mostly oriented along two axes. This angle roughly corresponds to the angle between the two in-plane $\langle$111$\rangle$ axes which are within the (110) plane, as depicted in Fig. 5c.

With the magnetization direction determined within each magnetic domain, the different domain walls can now be identified. In addition to several 180$^\circ$ domain walls, there also several 71$^{\circ }$, 109$^{\circ }$ domain walls. The walls that show Bloch lines (i.e. show different orientation of the magnetization in different segments, as the one in the lower right part of Fig. 4b) in the area explored are the 180$^\circ$ domain walls. The Bloch points are observed with both chiralities. All the walls are quite straight. Depending on the wall type, we observe a slight increase in width, from (140±60) nm (DW71) to (210±60) nm (DW180) (the pixel size in the images is 30 nm, comparable to the instrument resolution in PEEM mode). The domain wall width is determined by fitting a sigmoid function to the extracted intensity profile (see Fig. 5d). This wall width is larger than the width observed in other magnetite surfaces, such as for the (111) surface, which was reported in Ref.^61^ to be 80 nm. In the areas under observation, we saw the magnetization in the domains always aligned along the two easy axis of bulk magnetite that are within the (110) surface. It is thus likely that we are observing a combination of bulk domains with orientations of easy axes within the surface with surface closure domains of the bulk magnetization, originating from domains oriented along easy axes which are not within the surface plane.

## Discussion

The Fe$_{3}$O$_{4}$ surface has been examined using a combination of LEEM, LEED, XAS and XMCD. Structural characterization performed by LEED and LEEM reveals that by cycles of sputtering and subsequent annealing in vacuum a well-ordered surface with the (1$\times$3) reconstruction is obtained. Both XAS and XMCD PEEM characterization results correspond to magnetite with Fe$^{2+}$ and Fe$^{3+}$ cations located in octahedral and tetrahedral sites within the crystal structure, although the surface seems to have an excess of Fe$^{3+}$. On the basis of XAS and XMCD spectra we were able to estimate an (effective) spin magnetic moment 3.4 $\mu _{B}$ and an orbital magnetic moment of 0.6 $\mu _{B}$. Given that XMCD predominantly probes the near-surface layers, it is suggested that the decreased spin magnetic moments relative to the expected bulk value stem from the reconstructed surface. A significant dichroism is observed at the oxygen K-edge, at energies corresponding to the oxygen *p* bands hydridized with the *d* bands from the iron cations. The dichroism allows to observe the magnetic domains also at the oxygen edge, although the contrast is reversed relative of the octahedral iron signal. From XMCD PEEM images captured at different azimuthal angles at room temperature we have determined the vector magnetization of the surface, observing domains aligned along the in-plane $\langle$111$\rangle$ directions, with straight domain walls between them.

## Methods

Experiments were conducted at the CIRCE-XPEEM beamline of the Alba Synchrotron Light Facility^62^ and the DEMETER-PEEM end-station at the SOLARIS synchrotron^63^. Both microscopes can operate in pure Low-Energy Electron Microscopy (LEEM) mode, allowing in addition to determining the real space surface morphology also reciprocal space imaging (micro-spot Low-Energy Electron Diffraction or $\mu$LEED). Using synchrotron light, they can be used as (X-ray) PhotoEmission Electron Microscopes (XPEEM), enabling the acquisition of images of the energy-filtered photoelectron distribution from micron-sized selected areas of the surface, with an energy resolution below 0.2 eV. The sample is illuminated by incoming photons at an angle of 16$^\circ$ from the surface plane, while the sample azimuthal direction relative to the x-ray beam can be adjusted. The kinetic energy of the photoelectrons used to form the image can also be selected. Additionally, X-ray Absorption Spectroscopy (XAS) images can be obtained, as well as dichroism images from the pixel-by-pixel difference (asymmetry) of pairs of images recorded with opposite x-ray polarization directions^64,65^. We acquire the x-ray absorption images by selecting secondary electrons with a kinetic energy of typically 2 eV.

The magnetite crystal was supplied by SurfaceNet GmBH. A hat-shaped form was prepared with a polished top part of the hat. This design helps to avoid any strain on the top surface and simplifies the preparation process, as Fe$_{3}$O$_{4}$ crystals tend to be very brittle. Furthermore, the hat space give a more flat equipotential surface so the crystal can be explored without changing the tilt setting of the manipulator. The Fe$_{3}$O$_{4}$ (110) surface was cleaned following the method described by Jansen et al.^27^. Initially, the crystal was sputtered with Ar$^{+}$ ions at 1 keV energy, followed by successive annealing. The annealing process was carried out using electron beam heating, with the temperature varying from 850 K to 1200 K, checking the diffraction pattern in LEED mode. During the sample’s annealing, the total pressure in the preparation chamber was kept at 1$\times$10$^{-9}$ mbar at the maximum heating temperature of 1200 K.

## Data Availability

The datasets used and/or analysed during the current study available from the corresponding author on reasonable request.
